# Dr. Cappe, on the Cow-Pox

**Published:** 1800-11

**Authors:** Robert Cappe

**Affiliations:** York


### To Dr. BATTY.

Dear Sir,

If it ſuit your convenience, pray inſert the incloſed Reſolutions of the Directors of the York Diſpenſary in the next Number of your Journal. I alſo ſend you two letters that preceded the meeting at the Diſpenſary, which you may diſpoſe of as you think proper; they will ſhew you, that we are not totally negligent of the welfare of our fellow-citizens, though ſo far remote from the centre of animation.

I am your affectionate friend,

*York, Oct.* 13, 1800. R. CAPPE.

---

### YORK DISPENSARY.

At a meeting of the Directors of the York Diſpenſary, held at the Merchants' Hall, in Foſsgate, on Thurſday, October 9, 1800, (Mr. Alderman Wilson in the Chair,)

Reſolved,

1. That it appears, from the opinion of many of the moſt experienced medical men, that a General Inoculation for the

Cow-Pox throughout the island of Great Britain and Ireland, would nearly effect the extinction of the casual Small-Pox, and consequently be the means of annually saving the lives of upwards of 45,000 children and grown persons.

2. That the Physicians and Surgeons belonging to the Dispensary be requested to form themselves into a society, for the purpose of inoculating all such persons as may present themselves for Inoculation for the Cow-Pox; and that the Society, so formed, do invite into their body such Physicians, Surgeons, and Apothecaries as may be resident in York, and who, in their opinion, may promote the objects of that Society.

3. That the Physicians and Surgeons of the Dispensary be desired to supply the country practitioners with Matter, when requested, it being found, from unquestionable authority, that it does not lose its active powers by being transported to the distance of several hundred miles.

4. That the Directors shall use their utmost endeavours to encourage Inoculation for the Cow-Pox in all the neighbouring villages, as an auxiliary means of preventing the introduction of the casual Small-Pox into this City.

5. That a public Subscription be solicited, for the purpose of defraying the expence that may be incurred by this new Institution; and that the same be applied by the Directors of the Dispensary in such a way as they may deem most likely to further the objects thereof.

6. That this plan shall be carried into execution with all convenient speed, with a view to obviate the dreadful effects of the casual Small-Pox, which at this time are attended with considerable mortality in many parts of the country.

7. That the Resolutions and Regulations of this Meeting be sent to all the Clergymen in York, and the neighbouring villages, with request of the Meeting, that they will communicate the same to their parishioners, pointing out the advantages attending the Inoculation for the Cow-Pox in any way they judge most likely to forward the views of this Meeting.

8. That the Medical Gentlemen of the Dispensary be requested to draw up and print such Rules for the use of the poor, as may be necessary to regulate their conduct and attendance on the Dispensary during the period of Inoculation for the Cow-Pox.

9. That Books be opened at both the York Banks, where the smallest sums will be received, in support of a plan that has for its object the extinction of the casual Small-Pox from this city and neighbourhood; it being a fact, supported on the best authority, that a body once made the subject of the Cow-Pox, is rendered incapable of receiving the infection of the Small-Pox.

10. That

10. That the Refolutions of this Meeting be figned by the Chairman, be printed for general diftribution, and publifhed in the three York Papers.

11. That the Thanks of this Meeting be given to the Chairman, for his great attention in conducting the bufinefs of the day.

THOMAS WILSON, Chairman.

---

TO the EDITOR of the YORK HERALD.

Sir,

IT is known probably already to many of your readers, that more than two years ago Dr. JENNER, a phyfician of long experience and high reputation in Gloucefterfhire, publifhed a defcription of a diforder, much lefs fevere than the Small-pox, which renders the human body permanently fecure againft the fmall-pox contagion. The fact was known many years ago, in fome parts of the ifland, among thofe who had received the difeafe cafually; was long the object of Dr. Jenner's attention; and, fince his firft publication, has been verified by the obfervation of many eminent phyficians and furgeons in London, and different parts of the country, and by many gentlemen not engaged in the profeffion of medicine. The difeafe has alfo been tranfplanted to the Continent with the fame good effect; and has been received by fome of the firft families in this kingdom. In London, an Inftitution for the inoculation of it has been eftablifhed, the care of which is committed to Dr. Pearfon,* to whofe active zeal the world is indebted for much of the early and far-extended intelligence refpecting this difcovery.

The progrefs of the obfervations on this difeafe, I have watched with much jealous attention; but at length my doubts have been removed, by evidence of the utmoft credibility: and having found Mr. Edward Wallis of the fame fentiments as myfelf, I have lately, with his obliging affiftance, inoculated fome children in this city with the newly difcovered difeafe; the firft, I believe, who have undergone the operation here. We have found the difeafe in every refpect correfpond with accounts already publifhed; and, indeed, as a friend of mine, an eminent furgeon in London, expreffed himfelf in a letter to

---

* The medical eftablifhment of this inftitution has lately been increafed; there are now three phyficians and three furgeons, befides confulting furgeons and vifiting apothecaries.

me a few days ſince, " we have not ſeen any indiſpoſition attend it worthy of notice."

To ſhow the advantages which the new inoculation promiſes, I will ſtate conciſely the peculiarities of the ſmall-pox and of this diſeaſe.

The ſmall-pox are communicated through the air; and the contagion attaching itſelf to clothes, merchandize, and letters, is ſpread over a vaſt extent of country, and is ſometimes conveyed acroſs the ſea to diſtant kingdoms. The ſmall-pox were brought from the Eaſt to Europe; they were in Britain probably before the birth of Mahommed. The Europeans have ſince ſpread this dreadful diſorder from the Equator to the Pole. The Danes carried it to Greenland; the Spaniards poured its ravages, more terrible than their arms, through the unhappy country of Peru: in the ſingle province of Quito, 100,000 perſons died of the ſmall-pox when they firſt appeared in that country.

The ſmall-pox are now, it is true, a much leſs dreadful diſtemper than formerly. The improved medical treatment has rendered the caſual or natural ſmall-pox much more mild; and inoculation ſtill farther diveſts this diſeaſe of its terrors. Still, however, ſome die of ſmall-pox in all ſeaſons, and in ſome ſeaſons many. From the London Bills of Mortality, it appears that the ſmall-pox have, upon an average, annually deſtroyed more than 2020 perſons during ſeventy-five years, ending at 1777; the total amount is 151,570. Since that date I have not the bills before me; but in 1798, 2237 died of ſmall-pox, and in 1799, 1111. The deſtruction made by ſmall-pox is probably ſtill more dreadful, ſince thoſe bills do not include the deaths of perſons buried out of London, or in grounds not belonging to the Eſtabliſhed Church; nor do they include the two populous pariſhes of Pancras and Mary-le-Bonne, in which the annual burials for ten years, ending in 1742, were 1041.* Still the number of deaths from ſmall-pox in London, great as it is, probably bears a leſs proportion to the deaths occaſioned by ſmall-pox through the whole iſland, than the population of London bears to the population of the whole iſland. —For, by the Bills of Mortality, it appears, that one fourth of all the deaths in London is of ſtrangers; who, it is diſcovered from the ſame ſource, do not fix in London till the age of 18 or 20; and therefore among the new ſettlers, it is probable, there are few who have not had the ſmall-pox previous to their arrival in London;—and many children born in London, have

* The Small-pox Hoſpital and the Foundling are in theſe Pariſhes.

have the ſmall-pox in the country.* The very *means* that ſave the lives of many who have this diſeaſe, increaſe the number of thoſe who receive it; and it is now annually fatal to a greater number of perſons than before the improvement in the treatment of it; and the increaſe of deaths is not merely in proportion to the increaſe of population. Free admiſſion to the open air, though neceſſary for the ſick, expoſes thoſe who have not had the diſeaſe, not merely to the chance, but almoſt to the certainty of taking the infection. Very few of the inhabitants of this iſland now eſcape it.

The ſmall-pox are ſevere and dangerous in proportion to the number of puſtules; and of 1000 perſons, inoculated for that diſeaſe, generally more than 800 have puſtules. The ſmall-pox are peculiarly dangerous to infants and very young children, and women in pregnancy, who very rarely eſcape abortion when they receive this diſeaſe caſually. "The natural ſmall-pox in pregnant women is fatal in at leaſt nineteen out of twenty caſes to the fœtus in the womb, and to three-fourths or four-fifths of the women:"† and of thoſe pregnant women, whom perſonal ſafety has compelled to ſubmit to inoculation, many have loſt their own lives, and very few have borne living children.

The newly diſcovered diſeaſe has been diſtinguiſhed by the name of *Cow-pox* or *Vaccine-pox*, having been originally obſerved on the teats of cows, in ſome of our dairy counties. It is never communicated but by contact, and then only when the fluid matter lies on the broken ſkin.

Many women in pregnancy have paſſed through this diſeaſe, and none have ſuffered from it.

Inſtead of being peculiarly dangerous to young infants, it ſeems often to be peculiarly mild in them.

The diſorder is attended with very ſlight fever, which ſeldom laſts longer than a few hours. In ſome inſtances eruptions have appeared; but they had a very different courſe from thoſe of the ſmall-pox, ſeldom coming to ſuppuration, and leaving no marks on the ſkin. And even in thoſe inſtances in which an eruption at all reſembling the ſmall-pox has appeared, it is now the opinion of medical gentlemen, moſt experienced in the diſeaſe, that it has ariſen from expoſure to the effluvia of ſmall-pox.

The vaccine-pox are communicated by a ſcratch as ſlight as

* By an Act of Parliament paſſed in the year 1767, which we owe to the humanity of Mr. Hanway, all pariſh infants are ſent in three weeks after birth, into the country, to be nurſed for ſix years.

† Med. Commentaries, 1794.

as that ufed for inoculating the fmall-pox; but the difeafe is fo little infectious, that it is fometimes neceffary to make repeated attempts before the inoculation fucceeds. Around the fcratch a watery veficle is formed, and an eryfipelatous rednefs foon furrounds the whole. This fometimes fpreads extenfively, but with little inconvenience; and, when properly managed, it is eafily checked. The rednefs abates, and a hardnefs lefs extenfive fucceeds. The veficle becomes dry, a cruft is formed over it, and the whole gradually heals. In fome inftances the cruft feparates, and leaves a fpot unhealed like that of an iffue. In fome of the earlier inoculations, thefe fore places became troublefome; but, at that time, the proper treatment of the inoculated part was not difcovered. There is generally a flight fwelling in the armpit, but it foon fubfides without requiring medicinal applications.

I will only add the refult of the obfervations of a fingle phyfician, with which thofe of others perfectly coincide. Dr. Woodville, whofe attendance on the Small-pox Hofpital of London qualifies him peculiarly as a judge of the comparative feverity of the two difeafes, expreffes himfelf much in favour of that recently difcovered. " I have no doubt, that the inoculated cow-pox is as much milder than the inoculated fmall-pox, as the latter difeafe is milder than the cafual fmall-pox; nay, it feems to me, from the very benign form in which the vaccine-pock has of late invariably appeared, that it may be confidered as a difeafe perfectly harmlefs in its effects."*—— The cow-pox are not hitherto known certainly to have been fatal in any inftance. A little infant, under Dr. Woodville's care, died in convulfions on the feventh day after it was inoculated with vaccine matter; but convulfions frequently occur in infants, and are frequently fatal.

Dr. Woodville had, feveral months ago, inoculated 1000 people with the fmall-pox, who had previoufly had the inoculated cow-pox; not one of them received the infection. Many elderly people, who had received the cow-pox cafually, were inoculated for the fmall-pox after many years, one at the diftance of 53 years, but did not receive the infection. From Dr. Jenner's firft pamphlet, and a letter lately publifhed, addreffed to him by Wm. Fermor, Efq. I have made the following lift of perfons, who had had the cow-pox many years before, and did not receive the fmall-pox on inoculation. Many were often expofed to them in their own families when epidemic.

* Woodville's Obfervations on the Cow-pox, page 30. Publifhed July 15, 1800.

From

| From Dr. JENNER. | | The interval of years between the infection of cow-pox and inoculation of small-pox. |
|---|---|---|
| Joseph Merret - - - | 25 | |
| Sarah Portlock - - - | 27 | |
| John Phillips - - - | 53 | |
| Mary Barge - - - - | 31 | |
| Elizabeth Wynne - - | 38 | |
| William Stinchcomb - | 10 | |
| Hester Walkley - - | 26 | |
| From Mr. FERMOR. | | |
| William Tredwell - - | 3 | |
| Alban Collingridge - - | 4 | |
| Mr. Stevens * - - - | 4 | |
| Thomas Slater - - - | 6 | |
| Mr. H. Collingridge - | 10 | |

* He nursed his own family in the natural small-pox with impunity, 27 years after having had the cow-pox.

Many similar facts have been published by Dr. Pearson, and other writers on the cow-pox, but it seems unnecessary to adduce them, since only one person in twenty, according to the highest statement, is by natural constitution incapable of receiving the small-pox. Of these twelve, therefore, none ought from this cause to have escaped the infection; and there are so many millions of chances to one, that all could not escape, that it may be esteemed practically impossible. The security against the small-pox was therefore derived from the cow-pox, taken, in most of these instances, early in life. Dr. Woodville has not found those persons incapable of receiving the small-pox, to exceed the proportion of one in sixty, and they are equally exempt from the cow-pox.

Is it presuming too much from these facts to believe that a person who has had the cow-pox, will remain for ever secure against the small-pox?

If the cow-pox ever eradicate the small-pox, this cannot happen till some very distant period. The Chinese, it is to be feared, will long cherish this enemy to mankind with their indiscriminate prejudice in favour of old habits. The immediate advantages it offers are the following: When the small-pox are epidemic, (and in towns they are annually epidemic) young infants and pregnant women may be protected against a disease to them peculiarly dangerous, by one not at all unfriendly to them.

One person in a family may be secured against the small-pox, by inoculation for the cow-pox, without danger to others, when peculiar circumstances render it improper to inoculate the whole family; family; but the ſmall-pox cannot be given to one, without danger to all in that family who have not had them.

The cow-pox too, in proportion as it is a milder diſeaſe than the inoculated ſmall-pox, will, on that account, more extenſively than common inoculation, diminiſh the fatality of caſual ſmall-pox. But the new inoculation will in a far greater proportion prevent the ravages of caſual ſmall-pox, ſince common inoculation is ſometimes, in towns, undoubtedly the ſource whence ſmall-pox are ſpread among the people.

Inoculation for the vaccine-pox ſhould be made early in life; for if it be deferred till the ſmall-pox become epidemic, it is not always a certain preſervative. Some people, who had been previouſly expoſed to the contagion of ſmall-pox, had that diſeaſe, though inoculated with vaccine-matter, before any ſymptom of illneſs had appeared. Sometimes, however, the reſult has been more favourable.——The inoculation ſhould be made with the leaſt ſcratch that may be effectual, as the ſize of the ſucceeding veſicle depends much on the ſize of the ſcratch; and the ſmaller the veſicle, the more ſpeedy is the healing proceſs. The matter for future inoculation ſhould be taken in its moſt limpid ſtate: it has generally been found ſucceſsful when taken about the eighth day.

We have reaſon to be infinitely thankful to Providence for the means, now put into our power, of immediately checking the ravages of one of the moſt fatal plagues; and for the cheering hope of entirely exterminating that ſcourge from the face of the earth. With theſe ſentiments I feel it not leſs than a duty to lend my aid in ſpreading around the knowledge of the advantages which the vaccine inoculation offers us.

I am, Sir, yours, &c.

*York, Sept.* 5, 1800. ROBERT CAPPE.

---

To the Editor of the York Herald.

Sir,

The following Communication I have deferred for ſome weeks, from the hope of attaining more accurate information reſpecting the mortality of ſmall-pox in this city.

In a former letter which appeared in your paper on the 6th of September, intended as an introduction to this, I ſtated that the average of deaths from ſmall-pox, for ſeventy-five years, within the bills of mortality of London, had annually exceeded 2020. If the population within the bills of mortality may be ſtated at 1,000,000, which is probably not far from the truth, the the proportion is one death from fmall-pox in every 500 inhabitants. I remarked, that there is reafon to believe that the mortality from fmall-pox, through the whole ifland, is not in lefs proportion to the population: it probably far exceeds this eftimate. The inhabitants of Manchefter, Liverpool, and Chefter, were enumerated in the year 1773, the total amount was 78,271, and the annual deaths from fmall-pox, on an average, were 381; * therefore one perfon died of this difeafe, every year, in 205 $\frac{166}{381}$ of the inhabitants. The caufe of this difference of mortality from fmall-pox, in London and thefe towns, is in part to be explained by the number of new fettlers in London. To obviate all doubt of exceeding the truth, in my former letter I ftated the deaths of emigrants from the country as only one-fourth of all deaths in the bills of mortality; but, in fact, they have fallen little fhort of one-third. † There are no means of obtaining an accurate eftimate of the mortality from fmall-pox in a town, the population of which is, in part, fupported by emigration from the country; but the neareft approach to the truth is made by comparing the deaths from fmall-pox with the births. This method even is fubject to the error of giving too fmall a proportion, from the removal of children into the country before they undergo the fmall-pox. In London there is one death from this difeafe in each 6 ½ ‡ of all births; in Liverpool, one in 5 ½ ||. I have not been able to afcertain the mortality of fmall-pox in this city, for the difeafes are not mentioned in many of the regifters of burials; but from the moft accurate information I have obtained, it is probable that the deaths from fmall-pox have, many years, born a greater proportion to the number of inhabitants than in London. If they were equal, the annual deaths from this difeafe would probably be about 32: but I have, with little trouble, collected the names of twenty-one children who have died within the laft three months of fmall-pox; and a more ftrict inquiry, I fear, might much increafe the lift.

" The general diffufion of the fmall-pox affords a popular

---

* Haygarth's Sketch of a Plan to exterminate the cafual Small-Pox, vol. 1, p. 142.

† Price's Obfervations on Reverfionary Payments, vol. 1, p. 340, 5th edition.

‡ The annual medium of regiftered births in London for five years to 1768, was 16,291. Price's Reverf. Paym. vol. ii. p. 345. " The number of thofe who died of fmall-pox in the laft four years, on an average, is 2544." Dimfdale's Thoughts on General and Partial Inoculation, p. 44, publifhed 1776. The proportion at prefent is probably not very different.

|| Dr. Dobfon, quoted by Dr. Haygarth, Sketch, vol. i. p. 140.

prejudice that no civil regulation can control its progrefs; but authentic proofs of its extermination from Rhode Ifland, New England, and St. Helena, being produced, no great effort of wifdom is required to conclude, that, by the like methods, it might be exterminated from Great Britain."§ And we have an example before us, in the bofom of our own country. A fociety was formed at Chefter, in the year 1778, on the fuggeftion of Dr. Haygarth, for checking the mortality of fmall-pox, and for entirely excluding them as an epidemic difeafe. The efforts of this fociety had the moft happy fuccefs; and its experience proved, that the fmall-pox may be prevented raging among the people, in any country, by a few fimple rules, and at little expence. The Chefter plan required a periodical general inoculation for fmall-pox. This was attended with fome difficulty; and as the period for inoculation, on the whole, moft to be preferred, was every fecond year, there were occafional deviations from the rule, attended with fome danger of rendering the fmall-pox epidemic in the town. And fome perfons could not be prevailed on to receive by inoculation, a difeafe which, in the moft favourable circumftances, is not without danger, and might prove fatal. Thefe obftacles to the accomplifhment of a purpofe fo much to be defired, are now happily removed by the difcovery of the cow-pox, a difeafe in its nature mild, not contagious; feldom attended with any ficknefs, never with danger, and affording permanent fecurity againft the contagion of fmall-pox. The prefent time, therefore, is incomparably more favourable for the execution of fuch a plan, than the period when it was adopted with fuccefs at Chefter. No reftraint need be impofed refpecting the feafons of inoculating for the cow-pox, and a general inoculation, tho' on fome accounts defirable, may not be abfolutely neceffary.

The fmall-pox are chiefly deftructive to the poor; the rich, for the moft part, fecure their children from the cafual fmall-pox by inoculation: but this is done at the rifk of their lefs intelligent neighbours. Thus an operation, which confers an invaluable bleffing on the few intelligent individuals who adopt it, becomes injurious to the lefs intelligent part of the community. Humanity calls aloud on us to fubftitute a difeafe that brings fecurity to thofe who feek it, and fpreads danger to none. The poor need no inducement to adopt the cow-pox; the choice is now theirs, whether they will fecure to their family life and health, or leave their children the prey of a loathfome and deftructive plague, from the dreadful confequences of which

§ Haygarth, Vol. i. p. 164.

which death would often, to the laborious poor, be a defirable releafe.

This city, remarkable for its liberal fupport of all works of public utility, would probably abundantly fupply the expence attending the execution of a plan for preventing the fmall-pox being ever again epidemic in the town. The advantages gained by the city and its neighbourhood, by excluding the epidemic fmall-pox, could not be expreffed by any pecuniary eftimate. That difeafe, though, in general, it firft appears among the poor inhabitants, yet quickly fpreads, from incaution, to the houfes of their more opulent neighbours. But were a plan, fimilar to that of Chefter adopted, fecurity would prevail; opulent families might confidently relieve the diftreffed poor, without the dread of fuffering for their humanity; the rich might fecure their children againft a terrible diforder, without the dread of fpreading contagion among their poor neighbours; the induftrious labourer would efcape the vifitation of ficknefs, often to him the forerunner of poverty, family difcord, and extreme wretchednefs; and the inhabitants of the country would find a vifit to the city, attended with no more danger to their infant families than their accuftomed walks in the meadows.

I was encouraged to hope, by friends with whom I had fpoken on this plan, that it will be received here with approbation; I therefore requefted of Dr. Haygarth, now refident in Bath, advice refpecting the execution of the plan; and am encouraged by him to believe, that even if the cow-pox were not fubftituted for the fmall-pox, the rules of the Chefter fociety, where-ever they may be adopted, will be an effectual means of excluding the epidemic fmall-pox. But the cow-pox render all reftraint unneceffary; and will, in their natural progrefs, entirely eradicate the fmall-pox. In the mean time the rules of the Chefter fociety may protect us from the ravages of the cafual fmall-pox.

The firft great object may be attained, by the parifhes offering to thofe who may accept it, the gratis inoculation of their children; leaving to the choice of the parents, whether they fhall have the fmall-pox or the cow-pox. If the former be preferred, the parents muft engage to obferve the regulations; if the latter, no reftrictions are required.

A fociety fhould be formed, in addition, to pay, by fubfcription, the rewards to parents and friends for taking care of the health and fafety of their neighbours,* and for the purpofe of providing a conftant fupply of cow-pox matter.

* The few rules neceffary for conducting fuch a fociety, may be feen in Dr. Haygarth's account of the Chefter Society.

The general inoculation of this city would be attended with lefs difficulty than general inoculation in other more populous towns, as Leeds, Liverpool, and Manchefter, where it has been accomplifhed. The furgeons of the Difpenfary have already humanely offered their fervices to the poor of the city and neighbourhood, extending to them the advantages the cow-pox offer. The public is already indebted to them, and their affiftance may facilitate the execution of a plan I have here fketched out.

The following paragraph I tranfcribe from a letter, with which Dr. Haygarth lately favoured me.

"You probably know that thefe fpeculations (referring to his own writing) to exterminate the cafual fmall-pox, have commenced in Germany. About a year ago, along with a tranflation of the *Sketch* [the title of one of Dr. Haygarth's publications] I received feveral papers in German and French, explaining a medical affociation for this purpofe. On this bufinefs three hundred phyficians had delivered their opinions. Moft of the principal governments in Germany fupport this meafure. They promife to fend an account of their proceedings every year to every government of Europe. This bufinefs is chiefly promoted by Drs. Junker, Fauft, Lenz, &c. &c."

In another part of his letter, Dr. Haygarth fays, that "an introduction of the vaccine, ftill more than of the variolous inoculation, would effectually promote the great object of my publications."

Dr. Hunter obligingly allows me to add the following extract from a letter I received from him a few days fince. "A general inoculation for the cow-pox, holds out to the community at large a fecurity againft the cafual fmall-pox, in a manner that muft meet the approbation of all ferious and fober perfons. The endeavour to introduce a mild difeafe, in order to exterminate one of an alarming and dangerous nature, reflects great honour on you and the gentlemen of this city, who have voluntarily ftepped forward in the caufe of fo much humanity; and I beg leave to affure you and the other gentlemen, that I am ready to give my gratuitous affiftance when called upon as a profeffional man."

It was lately reported that David Ainfworth, a child two years old, had the fmall-pox fince he had the cow-pox. The report is unfounded, though he flept with his fifter during the whole time fhe was in the natural fmall-pox. The girl had been inoculated for the cow-pox at the fame time as her brother, but the infection did not take place in her.

I am, Sir, yours, &c.

